# ﻿A new species of the genus *Calamaria* H. Boie in F. Boie, 1827 (Squamata, Calamariidae) from Xishuangbanna, Yunnan Province, China

**DOI:** 10.3897/zookeys.1253.161412

**Published:** 2025-09-26

**Authors:** Tierui Zhang, Yuhao Xu, Tan Van Nguyen, Nikolay A. Poyarkov, Gernot Vogel, Xinge Wang, Song Huang

**Affiliations:** 1 The Anhui Provincial Key Laboratory of Biodiversity Conservation and Ecological Security in the Yangtze River Basin, College of Life Sciences, Anhui Normal University, Wuhu 241000, Anhui, China; 2 State Key Laboratory of Plateau Ecology and Agriculture, Qinghai University, Xining 810016, China; 3 The School of Medicine & Pharmacy, Duy Tan University, Da Nang, 550000, Vietnam; 4 Center for Entomology & Parasitology Research, Duy Tan University, Da Nang, 550000, Vietnam; 5 Department of Vertebrate Zoology, Lomonosov Moscow State University, Leninskiye Gory, GSP–1, Moscow 119991, Russia; 6 Society for South East Asian Herpetology, Im Sand-3, D-69115 Heidelberg, Germany

**Keywords:** *Calamaria synergis* sp. nov., morphology, Mountain Jinuo, phylogenetics, taxonomy

## Abstract

A new species of reed snake, *Calamaria synergis***sp. nov.**, is described based on two specimens collected from Mountain Jinuo, Jinghong City, Xishuangbanna Dai Autonomous Prefecture, Yunnan Province, China. The new species is distinguished from its congeners by a unique combination of morphological characters, including eight enlarged maxillary teeth; rostral higher than wide; prefrontal shorter than frontal and contacting the first two supralabials; mental not in contact with anterior chin shields; single preocular and postocular; four supralabials, with the 2^nd^ and 3^rd^ contacting the eye; five infralabials; five scales surrounding the paraparietal; ventrals 161–166; subcaudals 20–23, paired; dorsal scales reduced to six rows at the tail base and further to four rows near the terminal subcaudals; tail relatively short (6.6–9.2% of total length), abruptly tapering at the tip; dorsum blackish-brown with a distinct pale nuchal ring; two outermost dorsal scale rows pale khaki with upper margins darkened; ventral surface uniform pale khaki. Phylogenetic analysis of the mitochondrial cytochrome *b* gene places the new species as sister to *C. andersoni* and *C. yunnanensis*, from which it differs by an uncorrected *p*-distance of 8.7% and 7.9%, respectively. *Calamaria synergis***sp. nov.** is currently known only from tropical evergreen forests of Xishuangbanna at elevations around 1,050 m asl. We propose its conservation status as Data Deficient (DD) following the IUCN Red List categories.

## ﻿Introduction

The genus *Calamaria* H. Boie in F. Boie, 1827, commonly known as Southeast Asian reed snakes, currently comprises 69 valid species distributed from northeastern India and southern Japan to the Maluku Islands of eastern Indonesia (Inger and Marx 1965; Uetz et al. 2025). Of these, 18 species have been currently recorded from China and the Indochinese region (including Laos, Cambodia, Thailand, and Vietnam), namely: *C. abramovi* Orlov, 2009; *C. arcana* Yeung, Lau & Yang, 2022; *C. andersoni* Yang & Zheng, 2018; *C. buchi* Marx & Inger, 1955; *C. berezowskii* Günther, 1896; *C. concolor* Orlov, Nguyen, Nguyen, Ananjeva & Ho, 2010; *C. dominici* Ziegler, Tran & Nguyen, 2019; *C. gialaiensis* Ziegler, Nguyen & Nguyen, 2008; *C. jinggangensis* Cai, Jiang, Wu, Huang, Fei & Ding, 2023; *C. lovii* Boulenger, 1887; *C. lumbricoidea* Boie, 1827; *C. nebulosa* Lee, 2021; *C. pavimentata* Duméril, Bibron & Duméril, 1854; *C. sangi* Nguyen, Koch & Ziegler, 2009; *C. schlegeli* Duméril, Bibron & Duméril, 1854; *C. septentrionalis* Boulenger, 1890; *C. strigiventris* Poyarkov, Nguyen, Orlov & Vogel, 2019; *C. thanhi* Ziegler & Le, 2005; and *C. yunnanensis* Chernov, 1962 (Poyarkov et al. 2019, 2023; Cai et al. 2023; Liang et al. 2024; Uetz et al. 2025). Notably, six species have been described or revalidated within the past eight years, suggesting that the diversity and taxonomy of *Calamaria* in the montane regions of China and Indochina remain incompletely understood.

During recent fieldwork in Mountain Jinuo, Jinghong City, Xishuangbanna Dai Autonomous Prefecture, Yunnan Province, China, we collected two unidentified snake specimens assignable to the genus *Calamaria* based on the following diagnostic characters: a cylindrical, vermiform body; a short, thick tail; a head not distinct from the neck; small eyes with round pupils; eight subequal maxillary teeth, often modified in shape; incomplete upper head scalation typical of colubroid snakes, with internasal, loreal, and temporal scales always absent; four or five supralabials, the posterior-most broadly contacting the parietal; a large paraparietal scale behind the last supralabial; dorsal scales in 13 rows throughout the body, all smooth; a single, entire cloacal plate; and paired subcaudal scales (following Inger and Marx 1965; David et al. 2023). Subsequent morphological and molecular analyses revealed that these two individuals are distinct from all seven species of *Calamaria* currently known from China, as well as all other congeners reported from the Indochina region, based on a unique combination of morphological traits and significant genetic divergence. We herein describe these specimens as a new species of the genus *Calamaria*.

## ﻿Materials and methods

### ﻿Sampling

One adult male specimen and one juvenile male specimen of *Calamaria* were collected from Xishuangbanna Dai Autonomous Prefecture, Yunnan Province, China. These specimens were humanely euthanised with 0.7% tricaine methane sulfonate (MS-222) solution. Fresh liver tissue was extracted and immediately preserved in 95% ethanol for the subsequent molecular analysis. Specimens were preserved in 75% ethanol for permanent storage and deposited in Anhui Normal University Museum, Anhui, China (**ANU**). Sampling procedures involving live snakes were approved by the Animal Ethics Committee of Anhui Normal University and complied with the Wild Animals Protection Law of China.

### ﻿Molecular phylogeny

Total genomic DNA was extracted from preserved liver tissue with the OMEGA Tissue DNA Kit D3396 (Omega Bio-Tek, Norcross, GA, USA). A fragment of the mitochondrial cytochrome *b* (Cyt *b*) gene was amplified via Polymerase Chain Reaction (PCR) using the primer pair L14910 (5’-GACCTGTGATMTGAAACCAYCGTTGT-3’) and H16064 (5’-CTTTGGTTTACAAGAACAATGCTTTA-3’) (Burbrink et al. 2000). The double-stranded PCR products were sequenced by General Biosystems (Anhui) Corp. Ltd. (Chuzhou, China), and raw sequences were assembled using SeqMan in the DNASTAR software package (Burland 2000).

A total of 42 sequences from 14 known *Calamaria* species and three out-group species, including *Elaphe quatuorlineata* (Lacépède), *Orientocoluber spinalis* (Peters), and *Lycodon rufozonatus* Cantor, were obtained from GenBank or newly sequenced and incorporated into our dataset (see Table 1). DNA sequences were aligned by the Clustal W algorithm with default parameters (Thompson et al. 1997) and trimmed with gaps partially deleted in MEGA X (Kumar et al. 2018). Bayesian inferences (BI) were conducted in MRBAYES v. 3.2.7a (Ronquist et al. 2012) under the GTR + I + G model on Phylosuite v. 1.2.3 (Zhang et al. 2020; Xiang et al. 2023). In the BI analysis, three independent runs were conducted with 1 × 107 generations and sampled every 1,000 generations, with the first 25% of samples discarded as burn-in. In the ML analysis, the bootstrap consensus tree was inferred from 1000 replicates. Maximum likelihood (ML) was conducted under the best-fit substitution model (GTR + I + G) in RaxmlGUI 1.3 (Silvestro and Michalak 2012). Bootstrap proportions (BSP) were investigated with 1,000 bootstrap replicates using the fast-bootstrapping algorithm. Uncorrected pairwise genetic distances (*p*-distance) of the Cyt *b* gene among *Calamaria* species examined were calculated with MEGA X (Kumar et al. 2018).

**Table 1.** DNA sequences, voucher specimens, and GenBank accession numbers of the genus *Calamaria* and outgroup taxa used in this study.

Species	Specimen voucher no.	Locality	GenBank	Sources
*Calamaria synergis* sp. nov.	AHNU ZR24046	Mt. Jinuo, Xishuangbanna, Yunnan, China	PV745121	This study
*Calamaria synergis* sp. nov.	AHNU ZR25021	Mt. Jinuo, Xishuangbanna, Yunnan, China	PV745122	This study
*C. alcalai*	PNM 9873	Sitio Palbong, Barangay Batong Buhay, Sablayan, Mindoro, Philippines	MT819383	Weinell et al. 2020
*C. andersoni*	SYS r001699	Yingjiang, Yunnan, China	MH445955	Yang and Zheng 2018
*C. andersoni*	HS R20101	Dehong, Yunnan, China	OQ354844	Cai et al. 2023
*C. andersoni*	HS R20181	Tengchong, Yunnan, China	OQ354845	Cai et al. 2023
*C. andersoni*	AHNU ZR25021	Mangshi, Dehong, Yunnan, China	PV745123	This study
*C. arcana*	KFBG 14611	Mt. Dadongshan, Guangdong, China	ON482335	Yeung et al. 2022
*C. arcana*	HS 17082	Mt. Dawu, Guangdong, China	OQ354835	Cai et al. 2023
*C. arcana*	GP 9975	Yongxing, Hunan, China	OP980549	Cai et al. 2023
*C. arcana*	DL R199	Mt. Wuyi, Fujian, China	OQ354834	Cai et al. 2023
*C. berezowskii*	GXNU DLR194	Mt. Gongga, Sichuan, China	PP747047	Liang et al. 2024
*C. berezowskii*	GXNU DLR195	Mt. Gongga, Sichuan, China	PP747048	Liang et al. 2024
*C. berezowskii*	GXNU 20221215002	Mt. Gongga, Sichuan, China	PP747049	Liang et al. 2024
*C. gervaisii*	KU 324661	Puguis, La Trinidad, Benguet, Luzon, Philippines	MT819384	Weinell et al. 2020
*C. gervaisii*	KU 334485	Narvacan, Ilocos Sur, Luzon, Philippines	MT819385	Weinell et al. 2020
*C. jinggangensis*	DL 20200725	Mt. Jinggangshan, Jiangxi, China	OQ354830	Cai et al. 2023
*C. jinggangensis*	DL 20200625-2	Mt. Jinggangshan, Jiangxi, China	OQ354831	Cai et al. 2023
*C. jinggangensis*	DL 20200625-3	Mt. Jinggangshan, Jiangxi, China	OQ354832	Cai et al. 2023
*C. jinggangensis*	DL 20200625-4	Mt. Jinggangshan, Jiangxi, China	OQ354833	Cai et al. 2023
*C. lumbricoidea*	KU 315159	Pasonanca NP, Zamboanga, Philippines	MT819388	Weinell et al. 2020
*C. lumbricoidea*	KU 334479	Mt. Lumot, Gingoog, Misamis, Philippines	MT819389	Weinell et al. 2020
C. cf. lumbricoidea	USMHC 1560	Air Itam Dam, Penang, Malaysia	MN338526	Quah et al. 2020
*C. muelleri*	TNHC 58955	Gowa, South Sulawesi, Indonesia	MT819390	Weinell et al. 2020
*C. muelleri*	RMB 1283	Gowa, South Sulawesi, Indonesia	MT819391	Weinell et al. 2020
*C. nebulosa*	FMNH 258666	Phongsaly, Laos	MN338524	Quah et al. 2020
*C. palavanensi*	KU 309445	Barangay Irawan, Puerto Princessa, Palawan, Philippines	MT819386	Weinell et al. 2020
*C. palavanensi*	KU 311411	Mt. Mantalingahan, Rizal, Palawan, Philippines	MT819387	Weinell et al. 2020
*C. pavimentata*	KFBG 14507	Ningming, Guangxi, China	MH445957	Yang and Zheng 2018
*C. schlegeli*	LSUHC 10278	Bukit Larut, Perak, Malaysia	MN338525	Quah et al. 2020
*C. septentrionalis*	FTB 2839	unknown locality	KR814699	Pyron unpublished data
*C. septentrionalis*	KFBG 14506	Hainan, China	MH445956	Yang and Zheng 2018
*C. septentrionalis*	HS 11119 (CHS 116)	Tunxi, Huangshan, Anhui, China	MK201273	Li et al. 2020
*C. septentrionalis*	HS 12055 (CHS 118)	Huangshan, Anhui, China	MK201274	Li et al. 2020
*C. septentrionalis*	RE 30 (CHS 302)	Mangshan, Hunan, China	MK201384	Li et al. 2020
*C. septentrionalis*	SYS r000932 (CHS 613)	Guangdong, China	MK201434	Li et al. 2020
*C. septentrionalis*	HS R19100	Mt. Huangshan, Anhui, China	OQ354842	Cai et al. 2023
*C. septentrionalis*	HS 11145	Mt. Nanling, Guangdong, China	OQ354840	Cai et al. 2023
*C. septentrionalis*	DL 2021610-1	Huangsha, Guangxi, China	OQ354838	Cai et al. 2023
C. cf. septentrionalis	ROM 35605	Phia Oac-Phia Den NP, Cao Bang, Vietnam	AF471081	Lawson et al. 2005
C. cf. septentrionalis	ROM 35597	Phia Oac-Phia Den NP, Cao Bang, Vietnam	KX694890	Alencar et al. 2016
*C. yunnanensis*	ROM 41547	Simao, Yunnan, China	KX694891	Zaher et al. 2009
*C. yunnanensis*	YPx 503	Yunnan, China	JQ598922	Grazziotin et al. 2012
*C. yunnanensis*	QHU R2024054	Mt. Wanzhangshan, Simao, Yunnan, China	PV755783	This study
**Outgroup**				
*Orientocoluber spinalis*	MVZ 211019	Ningxia, China	AY486924	Nagy et al. 2004
*Elaphe quatuorlineata*	LSUMZ 40626	Hungary	AY486931	Nagy et al. 2004
*Lycodon rufozonatus*	LSUMZ 44977	China	AF471063	Lawson et al. 2005

### ﻿Morphological analysis

Terminology and measurements follow Inger and Marx (1965). Measurements were taken with a digital slide caliper to the nearest 0.1 mm, except for body and tail lengths, which were measured to the nearest 1.0 mm with a measuring tape. The number of ventral scales was counted according to Dowling (1951). The numbers of dorsal scale rows are given at one head length behind the head, at midbody, and at one head length before the vent. The sex was determined by dissection (inspection of gonads) and/or by tail shape when dissection was not possible. Maxillary teeth of the specimens were counted by examining both maxillae using a dissecting pin under a binocular microscope prior to preservation.

The following measurements (all in mm) and counts were taken: head length (**HL**, from snout tip to jaw angles) ; head width (**HW**) ; interorbital distance (**IOD**) ; eye-nostril distance (**EN**, from anterior edge of orbit to posterior edge of nostril) ; eye diameter (**EyeD**, horizontal) ; eye-mouth distance (**Eye-MouthD**, measured from the lowest point of the eye to the mouth gap) ; snout length (**SnL**, from the tip of rostral to the anterior margin of the eye) ; the number of dorsal scales reducing to six rows above the position of the subcaudal anterior to the tail tip (**DSR6R**) ; the number of dorsal scales reducing to four rows above the position of the subcaudal anterior to the tail tip (**DSR4R**) ; snout-vent length (**SVL**) ; tail length (**TaL**) ; total length (**TL**) ; ratio of tail length/total length (**TaL/TL**) ; dorsal scale rows number (**DSR**) ; supralabial scales (**SL**) ; number of supralabials touching the eye (**SL-E**) ; infralabial scales (**IL**) ; preocular scales **(PrO**) ; postocular scales (**PoO**) ; subcaudal scales (**SC**) ; ventral scales (**VEN**). Asymmetric characters are given in left/right order. Other abbreviations: Mt.: Mountain; NR.: Nature Reserve; NP.: National Park; Is.: Island; asl.: above sea level. For museum abbreviations, see Suppl. material 1: table S1.

Morphological and chromatic characters of the examined specimens were compared in detail to other species of the genus *Calamaria* known to occur in mainland Southeast Asia and other congeners. The examined comparative material is listed in Suppl. material 1: table S2. For comparison with other taxa, we relied on previously published data (Duméril et al. 1854; Inger and Marx 1965; Darevsky and Orlov 1992; Ziegler and Le 2005; Ziegler et al. 2007, 2008, 2019; Orlov 2009; Orlov et al. 2010; Yang and Zheng 2018; Poyarkov et al. 2019; Lee 2021; Yeung et al. 2022; Cai et al. 2023; Liang et al. 2024; Nguyen et al. 2025; Zhang et al. 2025).

## ﻿Results

### ﻿Molecular results

**Figure 1.**
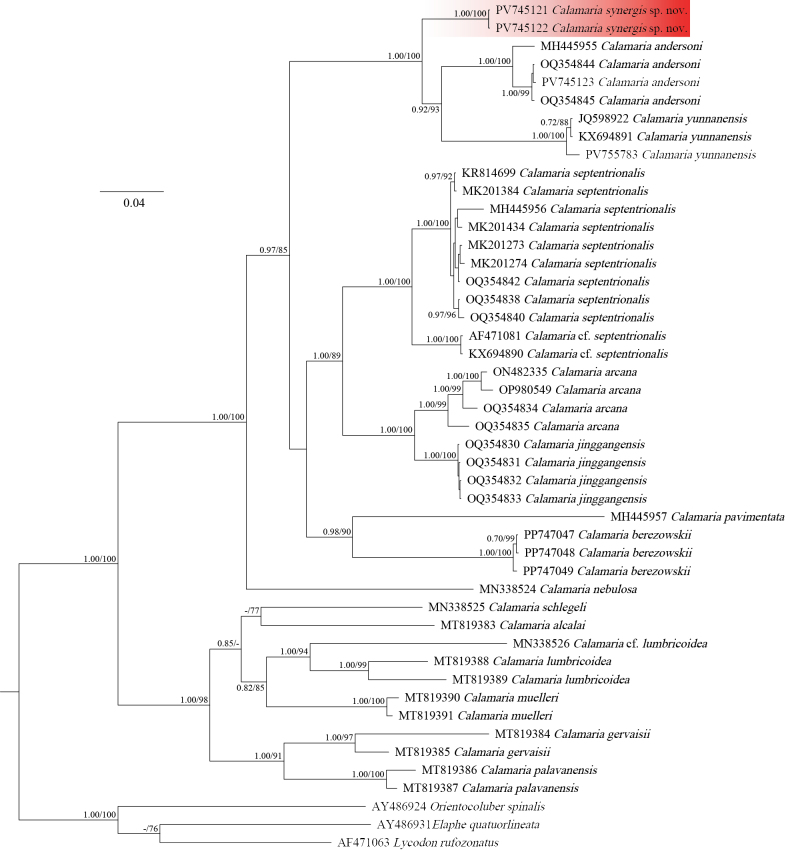
The Bayesian tree of *Calamaria* derived from analyses of Cyt *b* gene. Bayesian posterior probabilities (BPP) from BI analyses/ bootstrap supports (BSP) from ML analyses are listed beside the nodes, the ones lower than 0.70 or 75 are displayed as “-” or omitted.

A total of 1079 base pairs (bp) of the mitochondrial *Cyt b* gene were successfully sequenced and aligned, including two sequences from the putative new species. Both ML and BI analyses yielded congruent topologies (Fig. 1), which are consistent with those reported in previous studies (Cai et al. 2023; Liang et al. 2024). Within the current taxon sampling, the genus *Calamaria* was recovered as a monophyletic group with strong support (BPP = 1.00; BSP = 100). The two individuals representing *Calamaria* sp. from Xishuangbanna formed a distinct and well-supported clade (BPP = 1.00; BSP = 100), which was recovered as the sister lineage to a clade comprising *C. andersoni* and *C. yunnanensis* (BSP = 100; BPP = 1.00). Uncorrected *p*-distances between the Xishuangbanna *Calamaria* sp. and other congeners ranged from 7.9% (vs *C. yunnanensis*) to 21.6% (vs C. cf. lumbricoidea), with a p-distance of 8.7% from the closely related *C. andersoni* (see Table 2).

**Table 2.** Uncorrected *p*-distances (percentage) between *Calamaria* species based on 1079 base pairs from the mitochondrial gene Cyt *b*. Remark: The GenBank accession numbers are consistent with those provided in Table 1.

No.	GenBank	Species	1	2	3	4	5	6	7	8	9	10	11	12	13	14	15	16
1	PV745121	*Calamaria synergis* sp. nov.																
2	MT819383	*Calamaria alcalai*	20.8															
3	MH445955	*Calamaria andersoni*	8.7	21.8														
4	ON482335	*Calamaria arcana*	14.1	19.6	16.0													
5	PP747049	*Calamaria berezowskii*	16.5	21.1	16.2	15.3												
6	MT819384	*Calamaria gervaisii*	20.8	17.3	20.0	19.7	20.7											
7	OQ354830	*Calamaria jinggangensis*	14.7	20.5	16.3	5.7	14.6	19.2										
8	MN338526	Calamaria cf. lumbricoidea	21.6	16.5	21.2	20.4	19.9	18.6	20.9									
9	MT819389	*Calamaria lumbricoidea*	19.9	14.9	19.7	19.2	19.8	17.3	18.7	13.3								
10	MT819391	*Calamaria muelleri*	19.1	14.5	20.2	17.2	19.9	15.1	15.9	15.1	12.5							
11	MN338524	*Calamaria nebulosa*	16.4	21.4	17.2	16.4	16.6	21.0	16.5	20.0	18.4	18.0						
12	MT819386	*Calamaria palavanensis*	19.5	14.9	20.7	21.0	23.4	15.8	18.2	16.4	16.5	15.5	19.1					
13	MH445957	*Calamaria pavimentata*	16.8	21.8	18.6	17.0	15.8	22.0	15.7	21.5	20.4	21.8	17.7	22.2				
14	MN338525	*Calamaria schlegeli*	18.7	14.7	20.6	19.9	21.1	17.8	19.0	16.7	14.4	15.6	18.2	16.7	19.7			
15	MK201434	*Calamaria septentrionalis*	13.7	21.1	14.9	11.0	15.2	21.5	9.7	21.0	20.5	20.3	18.1	19.5	15.0	20.2		
16	KX694890	Calamaria cf. septentrionalis	14.2	21.7	14.8	10.6	14.2	21.2	10.4	20.1	20.1	20.2	17.1	20.1	16.6	19.8	4.4	
17	KX694891	*Calamaria yunnanensis*	7.9	21.7	9.8	16.3	17.1	22.0	14.9	21.2	20.6	22.3	16.9	22.2	17.7	20.6	14.9	14.2

Given the substantial genetic divergence and the presence of clear morphological differentiation from all known congeners (see the Comparisons section below), we recognise the examined specimens as representing a distinct previously unknown species, which we herein describe as a new species of the *Calamaria*.

### ﻿Taxonomic account

#### ﻿ Calamaria synergis sp. nov.

Taxon classificationAnimaliaSquamataCalamariidae

63A16D77-191C-55C8-986E-2628E872C376

https://zoobank.org/38C3599E-F659-45FA-B26C-DE28D0982DC2

**Figure 2.**
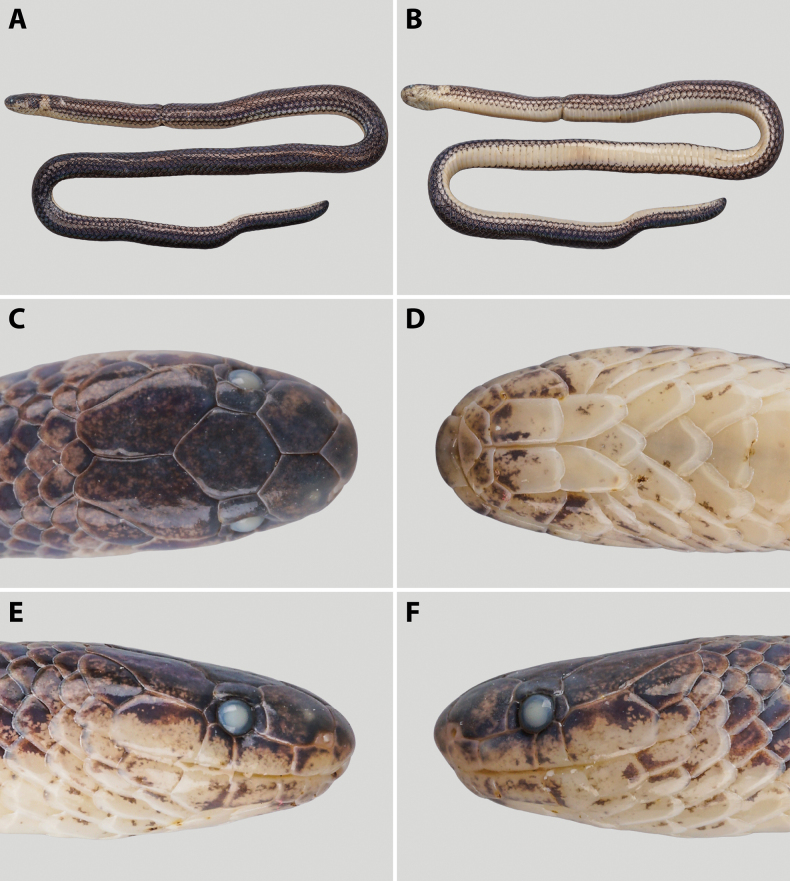
*Calamaria synergis* sp. nov. in preservative, holotype (ANU ZR24046, adult male). A. Dorsal view of body; B. Ventral view of body; C. Dorsal view of head; D. Ventral view of head; E. Right lateral view of head; F. Left lateral view of head. Photographs by TRZ.

**Figure 3.**
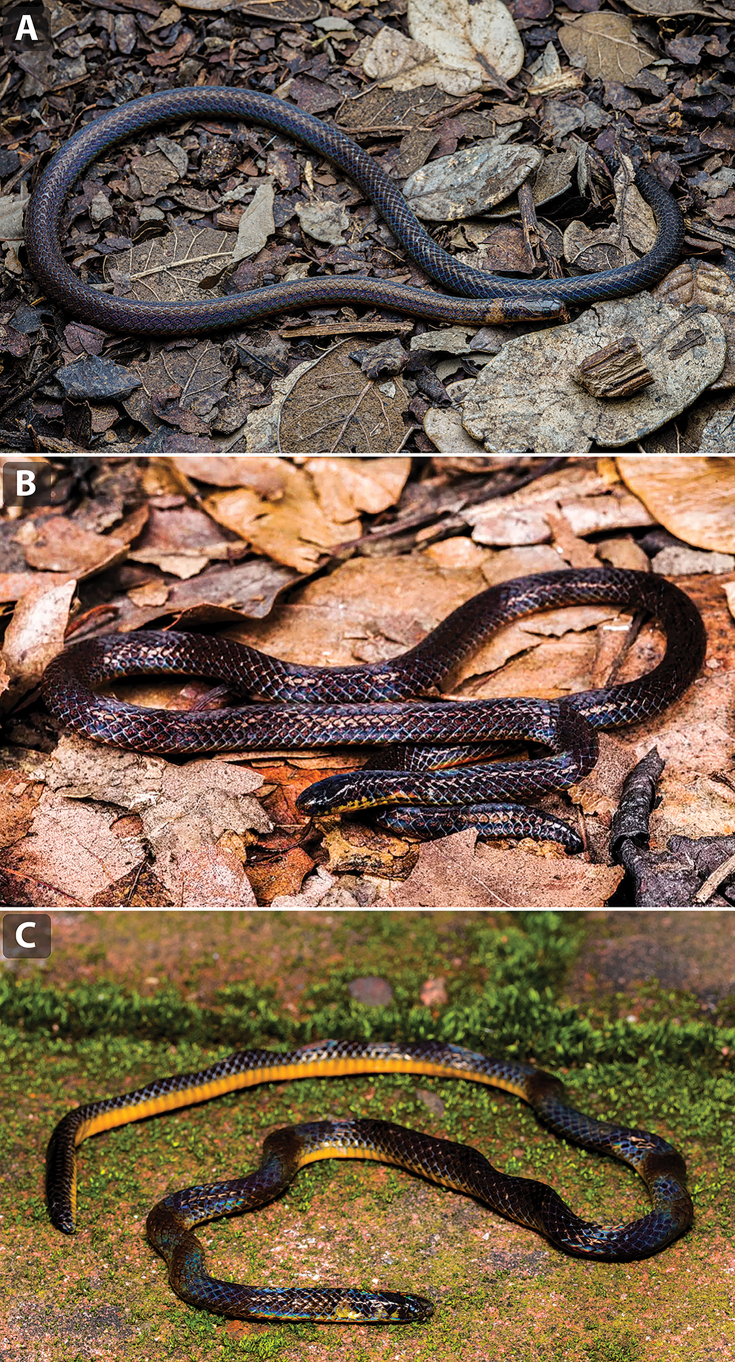
In situ photographs of *Calamaria synergis* sp. nov. and its congeners in life. A. *Calamaria synergis* sp. nov. (ANU ZR24046); B. *C. andersoni* (ANU ZR25022); C. *C. yunnanensis* (QHU R2024054). Photographs by TRZ (A, B) and T.Y. Zhang (C).

**Figure 4.**
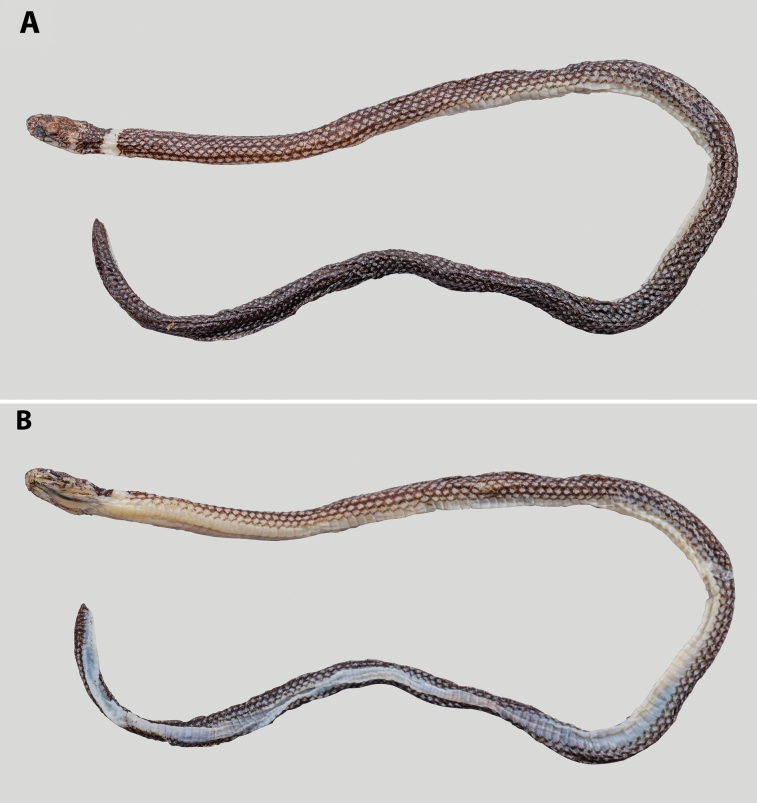
*Calamaria synergis* sp. nov. in preservative, paratype (ANU ZR25021, juvenile male). A. Dorsal view of body; B. Ventral view of body. Photographs by TRZ.

Figs 2, 3A, 4; Suppl. Figs S1A, S2A; Table 3

##### Type material.

***Holotype***: ANU ZR24046, adult male from Mountain Jinuo, Jinghong City, Xishuangbanna Dai Autonomous Prefecture, Yunnan Province, China (22.009245°N, 101.014120°E; elevation 1,050 m asl), collected on 5 June 2024, by TRZ. ***Paratype***: ANU ZR25021, one juvenile male from the same location as the holotype, collected on 7 August 2023, by TRZ.

##### Diagnosis.

*Calamaria synergis* sp. nov. can be distinguished from all other congeners by the following combination of morphological characters: eight enlarged maxillary teeth; rostral higher than wide; prefrontal shorter than frontal and contacting the first two supralabials; mental not in contact with anterior chin shields; single preocular and postocular; four supralabials, with the 2^nd^ and 3^rd^ contacting the eye; five infralabials; five scales surrounding the paraparietal; ventrals 161–166; subcaudals 20–23, paired; dorsal scales reduced to six rows at the tail base and further to four rows near the terminal subcaudals; tail relatively short (6.6–9.2% of total length), abruptly tapering at the tip; dorsum blackish-brown with a distinct pale nuchal ring; two outermost dorsal scale rows pale khaki with upper margins darkened; ventral surface uniform pale khaki.

##### Description of the holotype

**(Fig. 2).** Specimen ANU ZR24046 is in excellent condition. Body slender and cylindrical (SVL 258 mm, TL 284 mm); body thickness ~4.3–5.9 mm; tail not as thick as body, base of tail 3.6 mm thick. Tail short (TaL 26 mm, TaL/TL 9.2%); tail uniformly cylindrical in the anterior part, then abruptly tapering at tip; tip of tail obtusely pointed; head small, elliptical in dorsal view (HL 7.3 mm, HW 4.6 mm, HH 3.7 mm); eye small and round (EyeD 1.2 mm), larger than eye-mouth distance (Eye-MouthD 0.9 mm), ED/HL 16.4%.

Rostral higher than wide (width 1.4 mm, height 1.6 mm), portion visible from dorsal aspect almost equal to the length of the prefrontal suture; prefrontal (length 1.8 mm) shorter than frontal (length 3.1 mm), not entering orbit, in contact with 1^st^ and 2^nd^ supralabial; frontal hexagonal, longer (length 3.1 mm) than wide (width 2.4 mm), ~3.0 times maximum width (0.8 mm) of supraocular; paraparietal surrounded by five scales; preocular 1/1 (left/right, hereinafter), higher than wide, slightly higher than postocular, not as high as eye diameter; postocular 1/1, higher than wide; nasals small, barely surrounding nostrils, surrounded by the 1^st^ supralabial, rostral and prefrontal; supralabials 4/4, 2^nd^ and 3^rd^ entering orbit, 4^th^ largest, relative supralabial width 4>2>1>3; mental triangular, not in contact with the anterior chin shields; infralabials 5/5, the first three pairs touching anterior chin shields, the first pair meeting in the midline, 4^th^ largest; anterior chin shields longer than wide (length 2.1 mm, width 1.1 mm), pentagonal, meeting in the midline; posterior chin shields shorter than the anterior ones (length 1.6 mm), touching anteriorly and separated posteriorly by the first gular scales.

Dorsal scales in 13 rows throughout the body, reducing to six rows above the 7^th^ subcaudal and to four rows above the penultimate pair of subcaudals. Dorsal scales homogeneous in size and entirely smooth; vertebral row not enlarged. Ventrals 161. Anal plate undivided. Subcaudals 20, paired, smooth; terminal scale single and rigid.

##### Dentition.

Maxillary teeth modified (enlarged), eight on each side (8/8).

##### Colouration of the holotype in life

**(Fig. 3A).** Dorsum of head and upper parts of supralabials brownish black, scattered with indistinct white flecks; ventral surface of head pale khaki, with black speckles on the infralabials and anterior portion of the anterior chin shields. The dorsal surface of the body and the tail brownish black with iridescent white flecks distributed over most dorsal scales. Two outermost dorsal scale rows are slightly pale khaki, with upper margins partially darkened, forming two indistinct, narrow black stripes along each side of the body. A distinct pale khaki nuchal ring is present, extending dorsally from the level of the 2^nd^–4^th^ ventral scales, equivalent in width to two dorsal scale rows. No pale rings or blotches are present on the neck or tail. Ventral scales of the body and subcaudal scales of the tail are pale khaki, with black pigmentation on the outermost corners; small black flecks are present along the median line of the ventral tail surface, extending from the 2^nd^ subcaudal scale to the tail tip.

##### Colouration of the holotype in preservative

**(Fig. 2).** After nearly one year in 75% ethanol, the dorsum of the body and tail remains brownish black; white flecks on the dorsal scales and narrow black stripes along the flanks are still visible. The nuchal ring and ventral surfaces of the body have faded to greyish white.

##### Variation.

Specimen ANU ZR25021 (paratype), a juvenile male (see Fig. 4), has a smaller body size (SVL 127 mm, TaL 9 mm, HL 6.0 mm, HW 3.1 mm, EyeD 0.8 mm, Eye-MouthD 0.5 mm). Compared to the holotype, it exhibits a higher number of ventrals (VEN 166 vs 161), subcaudals (SC 23 vs 20), and lacks black flecks along the median line of the ventral tail surface (vs present in the holotype).

##### Etymology.

The specific name *synergis* is a Latin noun given in the apposition, derived from the Greek *synergos* (συνεργός), meaning “working together”. It emphasises that the resolution of the taxonomic status of the new species is the outcome of coordinated international scientific cooperation. The name is given in reference to both the collaborative effort involved in describing this new species and the broader need for joint action to address the complex taxonomic problems within the genus *Calamaria* in the future. We suggest the following common names: “Mountain Jinuo reed snake” (English), “基诺两头蛇” (Chinese), “Rắn mai gầm hiệp lực” (Vietnamese), and “Цзинхунская карликовая змея” (*Tszinhunskaya karlikovaya zmeya*, Russian).

##### Comparisons.

Comparative morphological data for the new species and the currently recognised members of the genus *Calamaria* from Indochina to southern China are presented in Table 3 and Suppl. material 1: figs S1, S2.

**Table 3.** Comparison of morphological characters of *Calamaria synergis* sp. nov. with those of congeners which occur in China and Indochina. **Symbols**: (1) = Supralabials; (2) = Supralabials contacting orbit; (3) = Preocular (1 = present, 0 = absent); (4) = Mental touching chin shields (1 = yes, 0 = no); (5) = Number of scales contacting paraparietal; (6) = VEN in males; (7) = VEN in females; (8) = SC in males; (9) = SC (females); (10) = max TL (mm) in males; (11) = max TL (mm) in females; (12) = TaL/TL (%) in males; (13) = TaL/TL (%) in females; (14) = Tail: tapering (2), slightly tapered (1), or not (0); (15) = End of tail; (16) = Dorsal colour; (17) = Ventrals colour. Notes: N/a: Not available; diagnostic characters (with respect to *Calamaria synergis* sp. nov.) are highlighted in bold.

Species	(1)	(2)	(3)	(4)	(5)	(6)	(7)	(8)	(9)	(10)	(11)	(12)	(13)	(14)	(15)	(16)	(17)	Sources
***C. synergis* sp. nov.**	4	2/3	1	0	5	161–166	n/a	20–23	n/a	284	n/a	6.6–9.2	n/a	0	obtusely pointed	blackish-brown	pale khaki, unspotted	This study
***C. abramovi***	4	2/3	1	0	**6**	159	174	**26**	20	**139**	482	**13.3**	7.1	**2**	**sharply pointed**	**uniformly black**	**yellow-orange spots**	Orlov et al. 2009
***C. andersoni***	4	2/3	1	0	**6**	164–172	186	20–23	n/a	**351**	312	8.8–9.2	5.8	0	obtusely pointed	brownish with faint narrow black lateral stripes	**bright orange to orange-yellow, unspotted**	Yang and Zheng 2018; Zhang et al. 2025
***C. arcana***	4	2/3	1	0	**6**	**170**–**176**	192	20–22	12	303	n/a	7.2–11.8	4.7	0	obtusely pointed	brownish	**orange-red, unspotted**	Yeung et al. 2022; Zhang et al. 2023
***C. berezowskii***	4	2/3	1	0	**6**	**149**–**155**	153–171	22–25	12–16	290	245	6.6–10.5	6.5–6.9	0	obtusely pointed	blackish-brown or brown	pale khaki or white	Inger and Marx 1965; Liang et al. 2024
***C. buchi***	4	2/3	1	**1**	5	n/a	221–236	n/a	13–14	n/a	466	n/a	3.9–4.1	1	obtusely pointed	blackish with small pale spots	**yellow, unspotted**	Inger and Max 1965; Ziegler et al. 2008
***C. concolor***	**5**	2/3	1	**1**	5	**209**	n/a	19	n/a	**578**	n/a	7.2	n/a	1	obtusely pointed	**uniformly orange-red**	**bright red, unspotted**	Orlov et al. 2010; Nguyen et al. 2025
***C. dominici***	4 or 5	2/3 or 3/4	1	1 or 0	**6**	n/a	174	n/a	17 or 18	n/a	421	n/a	6.2	1	obtusely pointed	**dark with irregular yellow blotches**	**dark with few yellow blotches & bands**	Ziegler et al. 2019
***C. gialaiensis***	4	2/3	1	**1**	5	**191**	n/a	23	n/a	**457**	n/a	8.1	n/a	0	**rounded**	**pale greyish-brown with a faint dark neck collar and a few dark blotches along the posterior vertebral region**	**yellowish beige, unspotted**	Ziegler et al. 2008
***C. jinggangensis***	4	2/3	1	**0**	**6**	**157**–**158**	179	20	12	260	364	**15**	3.6	0	obtusely pointed	brownish black	**dark orange**	Cai et al. 2023; Zhang et al. 2024
***C. lovii ingermarxorum***	4	2/3	**0**	**1**	**6**	**205**	n/a	23	n/a	**318**	n/a	7.4	n/a	0	blunt	**unspotted bluish-grey with pale spots on four lateral neck scales**	**dark gray, unspotted**	Darevsky and Orlor 1992
***C. lumbricoidea***	**5**	**3/4**	1	**1**	4 or 5	**144**–**196**	137–229	17–27	13–21	**498**	642	6.3–11.4	3.9–8.3	1	**sharply pointed**	**black with narrow cream or yellow rings; head red or pink in juveniles**	**yellow with black ventral scales forming bands**	Inger and Marx 1965
***C. nebulosa***	4	2/3	**0**	**0**	**6**	n/a	179	n/a	22	n/a	354	n/a	7.9	0	obtusely pointed	bluish-grey	**yellow, unspotted**	Lee 2021
***C. pavimentata***	4	2/3	1	**0**	n/a	**151**	n/a	**27**	n/a	n/a	n/a	n/a	n/a	**2**	**sharply pointed**	with narrow dark longitudinal stripes and a solid black collar behind the neck	yellowish white, unspotted	Duméril et al. 1854; Inger and Marx 1965
***C. sangi***	4	2/3	1	**1**	5 or 6	**190**	n/a	19	n/a	**373**	n/a	6.2	n/a	1	obtusely pointed	greyish-brown with fine dark mottling	**cream with narrow dark transverse bands**	Nguyen et al. 2009
***C. schlegeli***	**5**	**3/4**	1 or 0	**0**	5 or 6	129–161	136–180	**25**–**44**	19–37	**391**	395	**11.1**–**21.3**	7.3–14.4	0	**blunt**	**unspotted grey or brown; head variably pink, yellow, and/or brown**	cream, unspotted	Inger and Marx 1965
***C. septentrionalis***	4	2/3	1	**0**	**6**	148–166	168–188	**15**–**19**	6–11	**344**	384	6.3–8.6	2.6–4.3	0	**broadly rounded**	**dark brown or black dorsally, usually with a narrow yellow ring ~6**–**8 scales behind the head**	**yellow, with small black spots**	Inger and Marx 1965
***C. strigiventris***	4	2/3	1	**0**	**6**	130–168	176–183	**29**–**31**	20–22	**362**	367	**11.2**–**17.9**	8.4–0.6	**2**	**pointed**	**uniform grey-brown**	**bright yellow with longitudinal black stripes**	Poyarkov et al. 2019
***C. thanhi***	4	2/3	**0**	**0**	**6 or 7**	**184**	198	**28**	21	**461**	455	9.9	6.8	**2**	**gradually to a point**	**dark with 4**–**6 pale body bands**	**yellow, unspotted**	Ziegler et al. 2005, 2007
***C. yunnanensis***	4	2/3	**0**	**0**	**6**	**167**–**184**	189–199	15–21	16–19	296	516	5.4–8.4	5.0–5.5	**2**	obtusely pointed	blue-grey to olive-brown	**bright orange to orange-yellow, not spotted**	Lee 2021; Zhang et al. 2025

*Calamaria synergis* sp. nov. is readily distinguished from *C. lumbricoidea* (distributed in Thailand, Malaysia, Singapore, Indonesia, Brunei, and the Philippines) and *C. schlegeli* (Thailand, Malaysia, Indonesia, and Singapore) by having four supralabials, with the second and third in contact with the eye (vs 5 supralabials with the third and fourth in contact with the eye). Furthermore, both species are restricted to the areas south of the Isthmus of Kra in Peninsular Malaysia and have not been recorded from mainland Indochina.

The new species also differs from *C. lovii ingermarxorum* Darevsky & Orlov (Gia Lai Province, Vietnam), *C. nebulosa* (Phongsaly Province, Laos; possibly northern Thailand), *C. thanhi* (Quang Binh Province, Vietnam), and *C. yunnanensis* (Yunnan Province, China; possibly northwestern Vietnam) by the presence of a preocular scale (vs absent).

*Calamaria synergis* sp. nov. differs from *C. abramovi* (Kon Tum Province, Vietnam) in having the paraparietal surrounded by five shields and scales (vs six), fewer subcaudal scales in males (SC 20–23 vs 26), a maximum total length in males (max TL 284 mm vs 139 mm), shorter relative tail length in males (6.6–9.2% vs 13.3%), a non-tapering tail (vs gradually tapering), an obtusely pointed tail tip (vs sharply pointed), a blackish-brown dorsum (vs uniformly black), and pale khaki unspotted ventrals (vs yellow-orange spots).

Compared to *C. andersoni* (Fig. 3B; Suppl. material 1: Figs S1B, S2B), *Calamaria synergis* sp. nov. differs by having five shields surrounding the paraparietal (vs 6 in *C. andersoni*), a smaller maximum total length in males (max TL 284 mm vs 351 mm), eight maxillary teeth (vs 9), unspotted pale khaki ventrals (vs bright orange to orange-yellow), and the presence of light neck rings (vs absent).

The new species is distinct from *C. arcana* (Zhejiang, Guangdong, Fujian, and Hunan provinces, China) by five shields surrounding the paraparietal (vs 6), fewer ventrals in males (VEN 161–166 vs 170–176), unspotted pale khaki ventrals (vs orangish-red), and the presence of light neck rings (vs absent).

*Calamaria synergis* sp. nov. is further separated from *C. berezowskii* (Sichuan Province, China) by having more ventrals in males (VEN 161–166 vs 149–155), five shields surrounding the paraparietal (vs six), and the presence of light neck rings (vs absent).

The new species differs from *C. buchi* (Lam Dong Province, Vietnam) by the mentals not contacting the chin shields (vs contacting), fewer ventrals (161–166 vs 221–236), a blackish-brown dorsum (vs blackish with small pale spots), and unspotted pale khaki ventrals (vs yellow, unspotted).

From *C. concolor* (Thua Thien-Hue and Quang Nam provinces, Vietnam), the new species differs in having the mental not contacting the chin shields (vs contacting), fewer ventrals in males (VEN 161–166 vs 209), a smaller maximum total length in males (max TL 284 mm vs 578 mm), a non-tapering tail (vs slightly tapered), a blackish-brown dorsum (vs uniform orangish-red), unspotted pale khaki ventrals (vs bright red), and pale neck blotches (vs absent).

The new species is distinct from *C. dominici* (Dak Nong Province, Vietnam) in having five shields surrounding the paraparietal (vs 6), a non-tapering tail (vs slightly tapering), a blackish-brown dorsum (vs dark with irregular yellow blotches), and unspotted pale khaki ventrals (vs dark with few yellow blotches and bands).

Compared to *C. gialaiensis* (Gia Lai Province, Vietnam), the new species differs by the mental not contacting the chin shields (vs contacting), fewer ventrals in males (VEN 161–166 vs 191), a smaller maximum total length in males (max TL 284 mm vs 457 mm), an obtusely pointed tail tip (vs rounded), a blackish-brown dorsum (vs pale greyish-brown with a faint dark neck collar and posterior blotches), and unspotted pale khaki ventrals (vs yellowish beige).

*Calamaria synergis* sp. nov. can be distinguished from *C. jinggangensis* (Jiangxi and Guizhou, possibly Hunan provinces, China) by five shields surrounding the paraparietal (vs 6), slightly more ventrals in males (VEN 161–166 vs 157–158), shorter relative tail length in males (ratio TaL/TL 6.6–9.2% vs 15%), and unspotted pale khaki ventrals (vs dark orange).

The new species differs from *C. pavimentata* (Suppl. material 1: figs S1C, S2C), which is distributed from India and Myanmar through Indochina to Peninsular Malaysia and China (including Taiwan) and southernmost Japan, by having a higher number of ventrals in males (VEN 161–166 vs 151 in the holotype of *C. pavimentata*), fewer subcaudals in males (SC 20–23 vs 27 in the holotype), a non-tapering tail (vs tapered), and an obtusely pointed tail tip (vs sharply pointed).

*Calamaria synergis* sp. nov. is distinct from *C. sangi* (Kon Tum Province, Vietnam) by the mental not contacting the chin shields (vs contacting), fewer ventrals in males (VEN 161–166 vs 190), a smaller maximum total length in males (max TL 284 mm vs 373 mm), a non-tapering tail (vs slightly tapered), and the presence of neck blotches (vs absent).

The new species differs from *C. septentrionalis* (southern China and northern Vietnam) by the mental not contacting the chin shields (vs contacting), five shields surrounding the paraparietal (vs six), slightly more subcaudals in males (20–23 vs 15–19), a smaller maximum total length in males (max TL 284 mm vs 344 mm), an obtusely pointed tail tip (vs broadly rounded), a blackish-brown dorsum (vs dark brown or black, usually with a narrow yellow ring behind the head), and unspotted pale khaki ventrals (vs yellow with small black spots).

*Calamaria synergis* sp. nov. differs from *C. strigiventris* (Lam Dong and Khanh Hoa provinces, Vietnam) by the mental not contacting the chin shields (vs contacting), five shields surrounding the paraparietal (vs 6), slightly fewer subcaudals in males (SC 20–23 vs 29–31), a smaller maximum total length in males (max TL 284 mm vs 362 mm), a shorter relative tail length in males (ratio TaL/TL 6.6–9.2% vs 11.2–17.9%), a non-tapering tail (vs tapering), an obtusely pointed tail tip (vs abruptly pointed), a blackish-brown dorsum (vs uniform grey-brown), and unspotted pale khaki ventrals (vs bright yellow with longitudinal black stripes).

Finally, the new species differs from *C. yunnanensis* (see Fig. 3C, Suppl. material 1: figs S1D, S2D) by the presence of a preocular scale (vs absent), fewer ventral scales in males (VEN 161–166 vs 167–184), eight maxillary teeth (vs 9), unspotted pale khaki ventrals (vs bright orange to orange-yellow), and the presence of pale neck rings (vs absent).

##### Natural history, distribution, and conservation status.

**Figure 5.**
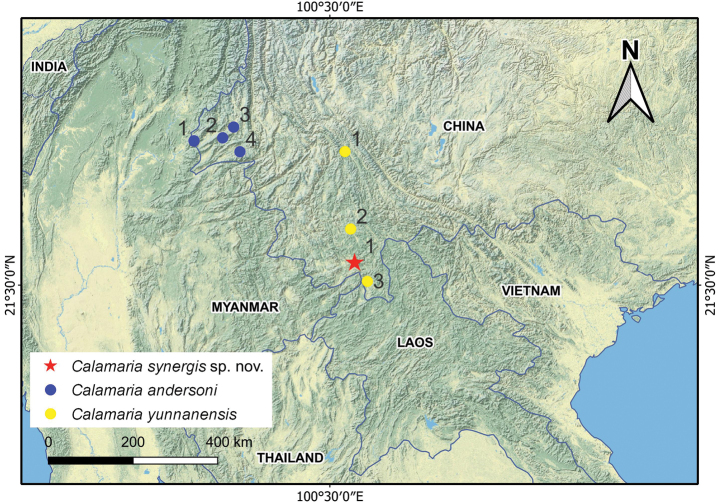
Distribution ranges of *Calamaria synergis* sp. nov., *C. andersoni*, and *C. yunnanensis*. Numbers indicate localities where the species have been recorded (see Suppl. material 1: table S3 for locality details).

*Calamaria synergis* sp. nov. is currently known only from tropical evergreen forest at its type locality (see Fig. 5), at an elevation of ca 1,050 m asl. This region is characterised by high herpetofaunal diversity, with several snake species occurring sympatrically in the same habitat, including *Oligodon fasciolatus* (Günther), *Lycodon fasciatus* (Anderson), *Plagiopholis nuchalis* Boulenger, and *Trimeresurus lanna* Idiiatullina, Nguyen, Pawangkhanant, Suwannapoom, Chanhome, Mirza, David, Vogel & Poyarkov (see also Idiiatullina et al. 2024; Vogel et al. 2024). It seems highly likely that the new species also occurs in the adjacent mountainous areas of eastern Myanmar, northern Laos, and northeastern Vietnam. Due to the limited information available on the ecology and potential threats of *Calamaria synergis* sp. nov., we recommend that the new species be classified as Data Deficient (DD) according to the IUCN Red List Categories and Criteria (IUCN 2025).

## ﻿Discussion

The discovery of *Calamaria synergis* sp. nov. brings the total number of recognised *Calamaria* species in China to eight: *C. andersoni*, *C. arcana*, *C. berezowskii*, *C. jinggangensis*, *C. pavimentata*, *C. septentrionalis*, *C. synergis* sp. nov., and *C. yunnanensis*. Among these, six species – *C. andersoni*, *C. arcana*, *C. berezowskii*, *C. jinggangensis*, *C. synergis* sp. nov., and *C. yunnanensis* – are currently considered endemic to China. Notably, the type locality of *Calamaria synergis* sp. nov. lies in close proximity to the borders of Myanmar, Laos, and Vietnam, suggesting the potential for a broader distribution encompassing Shan State (Myanmar), Phongsaly Province (northern Laos), and the northwestern provinces of Vietnam, such as Dien Bien, Lai Chau, and Lao Cai. These transboundary montane forests may represent a previously overlooked biogeographic corridor for regionally endemic fossorial taxa, and future surveys in these areas will be essential to delimit the species true distribution.

In Yunnan Province, China, four species of *Calamaria* are currently recorded: *C. andersoni*, C. cf. pavimentata, *C. yunnanensis*, and *C. synergis* sp. nov. (Yang and Rao 2008; Yang and Zheng 2018; this study). Among them, *C. synergis* sp. nov. exhibits several morphological similarities with *C. pavimentata*, a widespread species reported from multiple localities in Indochina and southern China. Yang and Rao (2008) reported populations of *C. pavimentata* from Jingdong (specimens KIZ 75II0225 [adult female] and KIZ 75II0230 [juvenile]) and from Fugong, Nujiang (KIZ 78II0007, adult female). The Jingdong specimens differ from *Calamaria synergis* sp. nov. in relative tail length (6.6–9.2% in males of *C. synergis* sp. nov. vs 5.5% in Jingdong females), ventral scale counts (161–166 in males vs 180–182 in females), and subcaudal scale counts (20–23 in males vs 14 in females). However, these differences fall within the expected range of sexual dimorphism in *Calamaria* (see Table 3), and the close geographic proximity of Jingdong to the type locality of *C. synergis* sp. nov. (ca 140 km) suggests that specimens KIZ 75II0225 and KIZ 75II0230 may be conspecific with *C. synergis* sp. nov. In contrast, the specimen from Fugong (KIZ 78II0007), also previously assigned to *C. pavimentata*, exhibits a relative tail length of 5.7%, 174 ventral scales, and 15 subcaudal scales, traits inconsistent with those of any currently recognised species in Yunnan Province. This individual may represent an undescribed species from the extreme north of the province. These findings highlight the need for a systematic re-evaluation of historical museum specimens, especially those from taxonomically ambiguous populations, using both morphological and genetic data to refine species boundaries and distribution records.

More broadly, our results underscore that species of *Calamaria* remain among the least understood snake lineages in mainland Southeast Asia. Their cryptic appearance, secretive habits, and strongly fossorial lifestyle make them particularly difficult to detect during field surveys. As a result, many species are known from very limited material, often a single specimen or a small series (e.g., Orlov et al. 2010; Yang and Zheng 2018; Lee 2021). This sampling bias likely contributes to an underestimation of the genus true diversity, especially in ecologically diverse but poorly explored montane zones of southern China and the Indochinese Peninsula.

To advance taxonomic knowledge and conservation assessments for this elusive group, targeted surveys using appropriate methods (e.g., systematic leaf-litter sifting, pitfall traps, and microhabitat-based searches) should be prioritised in mid-elevation evergreen forests across border regions. Furthermore, integrative approaches combining detailed morphology, molecular phylogenetics, and ecological niche modelling will be key to resolving complex species groups and identifying overlooked lineages. The sister-group relationship recovered between *Calamaria synergis* sp. nov., *C. andersoni*, and *C. yunnanensis* also suggests the existence of a regionally restricted clade within montane southwestern China, possibly shaped by shared ecological constraints or historical barriers to gene flow. Additional phylogeographic studies may help elucidate the origin, divergence, and evolutionary trajectories of this lineage within the broader context of *Calamaria* diversification.

The following key to the species of *Calamaria* known from China is adapted from Yeung et al. (2022), Cai et al. (2023), and Liang et al. (2024). It is provided as a practical reference for preliminary identification in the field or collection. However, given the morphological similarity among species and the presence of cryptic diversity within the genus, accurate species determination should be confirmed through detailed morphological examination and, where possible, supported by molecular data.

**Table:** 

1	Preocular present	**2**
–	Preocular absent	***Calamaria yunnanensis***
2	Dorsal scales reduced to 4 rows on the tail at last the subcaudals	**3**
–	Dorsal scales reduced to >4 rows on the tail at last the subcaudals	**6**
3	Pale rings or blotches on neck present	**4**
–	Pale rings or blotches on neck absent	***Calamaria andersoni***
4	Pale rings/blotches on tail present	**5**
–	Pale rings/blotches on tail absent	***Calamaria synergis* sp. nov.**
5	Tail tapering gradually to a point	***Calamaria pavimentata* complex**
–	Tail broadly rounded at tip (not tapering)	***Calamaria septentrionalis***
6	Less than half of posterior chin shields meeting at midline; indistinct nuchal ring or paired spots present	**7**
–	Posterior chin shields meeting extensively at midline; collar or nuchal spots absent	***Calamaria jinggangensis***
7	VEN 170–176 in males, VEN 192 in females	***Calamaria arcana***
–	VEN 149–155 in males, VEN 153–171 in females	***Calamaria berezowskii***

## Supplementary Material

XML Treatment for Calamaria synergis

## ﻿Additional information

### Use of AI

No use of AI was reported.

### Author ORCIDs

Tierui Zhang https://orcid.org/0009-0000-1869-9313

Yuhao Xu https://orcid.org/0000-0001-6094-6680

Tan Van Nguyen https://orcid.org/0000-0001-5413-968X

Nikolay A. Poyarkov https://orcid.org/0000-0002-7576-2283

Gernot Vogel https://orcid.org/0000-0002-4542-518X

Xinge Wang https://orcid.org/0000-0002-8938-1377

Song Huang https://orcid.org/0000-0001-6786-8523

### Data availability

All of the data that support the findings of this study are available in the main text or Supplementary Information.

## Supplementary materials

Supplementary material 1 Supplementary figures and tables Data type pdf Explanation note **fig. S1.** Comparative head shape and body coloration of *Calamaria synergis* sp. nov., *C. andersoni*, *C. pavimentata*, and *C. yunnanensis*. (A) *C. synergis* sp. nov. (ANU ZR24046); (B) *C. andersoni* (SYS r001699); (C) *C. pavimentata* (KFBG 14507); (D) *C. yunnanensis* (QHU R2024054). Each row shows: (1) dorsal view of head; (2) dorsal view of body; (3) dorsolateral view of body. Photographs by T.R. Zhang (A), reproduced from Yeung et al. (2022) (B, D), and Y.H. Xu (C). **fig. S2.** Comparison of tail shape and coloration of *Calamaria synergis* sp. nov., *C. andersoni*, *C. pavimentata*, and *C. yunnanensis*. (A) *C. synergis* sp. nov. (ANU ZR24046); (B) *C. andersoni* (SYS r001699); (C) *C. pavimentata* (KFBG 14507); (D) *C. yunnanensis* (QHU R2024054). Each row shows: (1) dorsal view of tail; (2) lateral view of tail; (3) ventral view of tail. Photographs by T.R. Zhang (A), reproduced from Yeung et al. (2022) (B, D), and Y.H. Xu (C). **table S1.** Museums, private collections and biorepository abbreviations mentioned in this study. **table S2.** List of specimens examined of *Calamaria*. **table S3.** List of localities of the *Calamaria synergis* sp. nov., *C. andersoni*, and *C. yunnanensis* appearing on Fig. 5. https://binary.pensoft.net/file/1424706This dataset is made available under the Open Database License (http://opendatacommons.org/licenses/odbl/1.0/). The Open Database License (ODbL) is a license agreement intended to allow users to freely share, modify, and use this Dataset while maintaining this same freedom for others, provided that the original source and author(s) are credited.Tierui Zhang, Yuhao Xu, Tan Van Nguyen, Nikolay A. Poyarkov, Gernot Vogel, Xinge Wang, Song Huang
