# Supplementary material for: ﻿A new species of the genus Calamaria H. Boie in F. Boie, 1827 (Squamata, Calamariidae) from Xishuangbanna, Yunnan Province, China
Source: Zookeys. 2025 Sep 26;1253:255–75. doi: 10.3897/zookeys.1253.161412 (PMC12495431; doi:10.3897/zookeys.1253.161412)
Supplement: Supplementary material 1 — Supplementary figures and tables [file zookeys-1253-255_article-161412__-s001.pdf]

A new species of the genus *Calamaria* H. Boie *in* F. Boie, 1827 (Squamata, Calamariidae) from Xishuangbanna, Yunnan Province, China

Nikolay A. Poyarkov[5], Gernot Vogel[6], Xinge Wang[1], Song Huang[1]

1 The Anhui Provincial Key Laboratory of Biodiversity Conservation and Ecological Security in the Yangtze River Basin, College of Life Sciences, Anhui Normal University, Wuhu 241000, Anhui, China

2 State Key Laboratory of Plateau Ecology and Agriculture, Qinghai University, Xining 810016, China

3 The School of Medicine & Pharmacy, Duy Tan University, Da Nang, 550000, Vietnam

4 Center for Entomology & Parasitology Research, Duy Tan University, Da Nang, 550000, Vietnam

5 Department of Vertebrate Zoology, Lomonosov Moscow State University, Leninskiye Gory, GSP–1, Moscow 119991, Russia

6 Society for South East Asian Herpetology, Im Sand-3, D-69115 Heidelberg, Germany

Corresponding authors: Tan Van Nguyen (tan.sifasv@gmail.com); Song Huang (snakeman@ahnu.edu.cn)

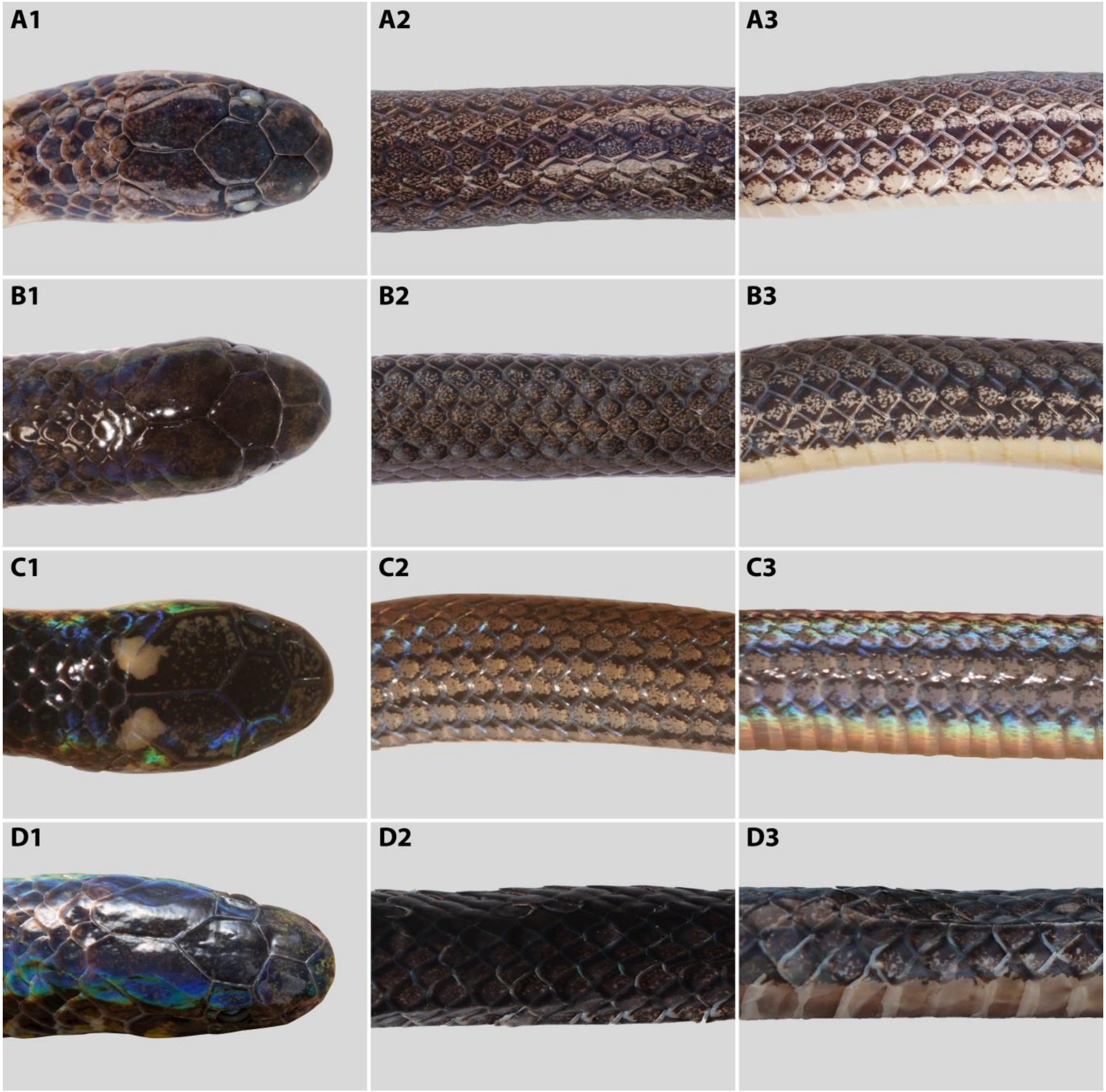

Suppl. Figure S1. Comparative head shape and body coloration of *Calamaria synergis* **sp. nov.**, *C. andersoni*, *C. pavimentata*, and *C. yunnanensis*. (A) *C. synergis* **sp. nov.** (ANU ZR24046); (B) *C. andersoni* (SYS r001699); (C) *C. pavimentata* (KFBG 14507); (D) *C. yunnanensis* (QHU R2024054). Each row shows: (1) dorsal view of head; (2) dorsal view of body; (3) dorsolateral view of body. Photographs by T.R. Zhang (A), reproduced from Yeung et al. (2022) (B, D), and Y.H. Xu (C).

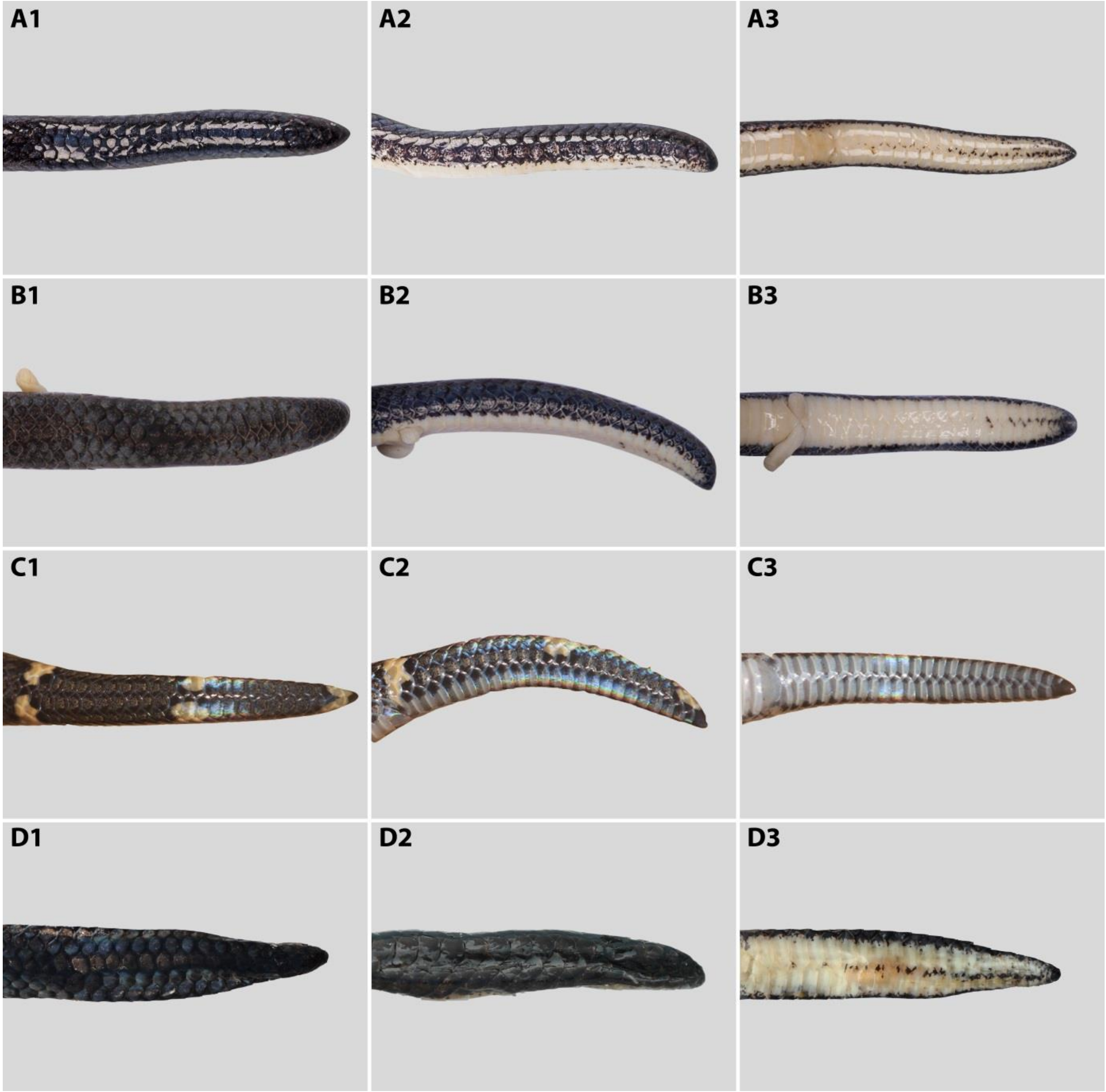

Suppl. Figure S2. Comparison of tail shape and coloration of *Calamaria synergis* **sp. nov.**, *C. andersoni*, *C. pavimentata*, and *C. yunnanensis*. (A) *C. synergis* **sp. nov.** (ANU ZR24046); (B) *C. andersoni* (SYS r001699); (C) *C. pavimentata* (KFBG 14507); (D) *C. yunnanensis* (QHU R2024054). Each row shows: (1) dorsal view of tail; (2) lateral view of tail; (3) ventral view of tail. Photographs by T.R. Zhang (A), reproduced from Yeung et al. (2022) (B, D), and Y.H. Xu (C).

Suppl. Table S1. Museums, private collections and biorepository abbreviations mentioned in this study.

ANU: Anhui Normal University, Wuhu, China
CAS: California Academy of Sciences, San Francisco, USA
DL: Ding Lee's private collection, Chengdu Institute of Biology, Sichuan, China
DTU: Duy Tan University, Da Nang, Vietnam
FMNH: Field Museum of Natural History, Chicago, USA
FTB: Frank T. Burbrink field tag series
GP: Guo Peng private collection, Yibin University, Yibin, China
GXNU: Guangxi Normal University, Guangxi, China
HS: Song Huang field tag series, Anhui Normal University, Anhui, China
KFBG: Herpetological Collection of Kadoorie Farm and Botanic Garden, Hong Kong, China
KIZ: Kunming Institute of Zoology, Yunnan, China
KU: Museum of Natural History, University of Kansas, Lawrence, USA
LSUHC: La Sierra University Herpetological Collections, Riverside, USA
LSUMZ: Louisiana State University Museum of Natural Science, Louisiana, USA
MHNG: Museum d'Histoire Naturelle, Geneva, Switzerland
MVZ: Museum of Vertebrate Zoology, University of California at Berkeley, Berkeley, California, USA
MZB: Laboratory of Herpetology at the Museum Zoologicum Bogoriense, Indonesian Institute of Sciences, Indonesia
MZMU: Departmental Museum of Zoology, Mizoram University, Mizoram, India
NHBE: Natural History Museum of Beijing, Beijing, China
NHMUK: The Natural History Museum, London, UK
NHMW: Naturhistorisches Museum Wien, Vienna, Austria
NMB: Naturhistorisches Museum, Basel, Switzerland
PNM: Philippine National Museum, Manila, Philippines
QHU: Qinghai University, Xining, China
RMB: Rafe M. Brown field tag (specimen deposited in Museum Zoologicum Bogoriense, Indonesia)
RMNH: Naturalis-Nationaal Natuurhistorisch Museum [formerly Rijksmuseum van Natuurlijke Historie], Leiden, Netherlands
ROM: Royal Ontario Museum, Toronto, Ontario, Canada
SMF: Naturmuseum Senckenberg, Frankfurt am Main, Germany
SYS: Sun Yat-Sen University, Guangzhou, Guangdong, China
THNHM: Thailand Natural History Museum, National Science Museum, Bangkok, Thailand;
TNHC: Texas Natural History Collections, University of Texas at Austin, Texas, USA
USMHC: Universiti Sains Malaysia Herpetological Collection, Penang, Malaysia
ZFMK: Zoologisches Forschungsmuseum Alexander Koenig, Bonn, Germany
ZMA: Universiteit van Amsterdam, Zoologisch Museum, Amsterdam, Netherlands
ZMB: Zoologisches Museum für Naturkunde der Humboldt-Universität zu Berlin, Berlin, Germany
ZMH: Zoologisches Museum Hamburg, Hamburg, Germany
ZMMU: Zoological Museum of Lomonosov Moscow State University, Moscow, Russia

Suppl. Table S2. List of specimens examined of *Calamaria*. See Suppl. Table S1 for abbreviations mentioned in sample IDs.

Species	**Type status**	**Specimen voucher**	**Locality**
C. albiventer	syntype of *Calamaria indragirica* Schenkel	NMB 1698	Indragiri, Riau, Sumatra, Indonesia
C. albiventer	lectotype of *Calamaria indragirica* Schenkel	NMB 1697	Indragiri, Riau, Sumatra, Indonesia
C. andersoni	holotype	SYS r001699	Tongbiguan, Yingjiang, Yunnan, China
C. andersoni		ANU ZR25022	Mangshi, Dehong, Yunnan, China
C. andersoni		ANU ZR24017	Mangdong, Lianghe, Dehong, Yunnan, China
C. andersoni		QHU 2025016	Mangdong, Lianghe, Dehong, Yunnan, China
C. andersoni		QHU 2025017	Mangdong, Lianghe, Dehong, Yunnan, China
C. andersoni		QHU 2025018	Mangdong, Lianghe, Dehong, Yunnan, China
C. andersoni		ANU HSR 21036	Mangdong, Lianghe, Dehong, Yunnan, China
C. bicolor	holotype	RMNH 68	Kalimantan, Borneo, Indonesia
C. bicolor		RMNH 10468	Kapuas River, Kalimatan, Borneo, Indonesia
C. bicolor		RMNH 1678	Borneo, Malaysia
C. bicolor		RMNH 10542	West coast of Borneo
C. bicolor	holotype of *C. picteti* Peracca	MHNG 748.036	Sarawak, Borneo, Malaysia
C. ceramensis	syntype	RMNH 10083A	Honitétu, Seram Is, Maluku, Indonesia
C. ceramensis	syntype	RMNH 10083B	Honitétu, Seram Is, Maluku, Indonesia
C. concolor	topotype	DTU 333	Bach Ma NP, Thua Thien-Hue, Vietnam
C. concolor	topotype	DTU 333	Bach Ma NP, Thua Thien-Hue, Vietnam
C. crassa	holotype	RMNH 4692	Mt. Talamau, Pasaman, West Sumatra, Indonesia
C. linnaei		RMNH 32a	Java, Indonesia
C. linnaei		RMNH 27	Java, Indonesia
C. linnaei		SMF 106775	Cibodas, West Java, Indonesia
C. linnaei		SMF 106777	Cibodas, West Java, Indonesia
C. linnaei		RMNH 7464B	Bandung, West Java, Indonesia
C. linnaei		RMNH 46513	Bogor, West Java, Indonesia
C. linnaei		RMNH 46514	Bogor, West Java, Indonesia
C. linnaei		RMNH 14488	Wonosobo, Central Java, Indonesia
C. linnaei		RMNH 14842	Garut, West Java, Indonesia
C. linnaei		SMF 106798	Bogor, West Java, Indonesia
C. linnaei		SMF 106795	Cibodas, West Java, Indonesia
C. linnaei		SMF 106794	Cibodas, West Java, Indonesia
C. linnaei		SMF 106776	Cibodas, West Java, Indonesia
C. linnaei		RMNH 7464A	Bandung, West Java, Indonesia
C. linnaei		RMNH 46517	Bogor, West Java, Indonesia
C. linnaei		RMNH 35605	Bogor, West Java, Indonesia

C. linnaei		RMNH 14456	Bandung, West Java, Indonesia
C. linnaei		SMF 106798	Bogor, West Java, Indonesia
C. linnaei		SMF 106795	Cibodas, West Java, Indonesia
C. linnaei		SMF 106794	Cibodas, West Java, Indonesia
C. linnaei		SMF 106775	Cibodas, West Java, Indonesia
C. linnaei		SMF 106776	Cibodas, West Java, Indonesia
C. linnaei		SMF 106777	Cibodas, West Java, Indonesia
C. linnaei		RMNH 7464A	Bandung, West Java, Indonesia
C. linnaei		RMNH 7464B	Bandung, West Java, Indonesia
C. linnaei		RMNH 46513	Bogor, West Java, Indonesia
C. linnaei		RMNH 46514	Bogor, West Java, Indonesia
C. linnaei		RMNH 46517	Bogor, West Java, Indonesia
C. linnaei		RMNH 35605	Bogor, West Java, Indonesia
C. linnaei		RMNH 14488	Wonosobo, Central Java, Indonesia
C. linnaei		RMNH 14842	Garut, West Java, Indonesia
C. linnaei		RMNH 29	Java, Indonesia
C. linnaei	syntype of *C. maculosa* Reinwardt in Boie	RMNH 32b	Java, Indonesia
C. linnaei complex	holotype *C. boschei* Roux	RMNH 8620	Tlogojati, Wonosobo, Central Java, Indonesia
C. linnaei	holotype of *C. reticulata* Reinwardt in Boie	RMNH 29	Java, Indonesia
C. linnaei	syntype of *C. maculosa*	RMNH 32a	Java, Indonesia
C. linnaei	syntype of *C. maculosa*	RMNH 32b	Java, Indonesia
C. linnaei	holotype	RMNH 27	Cihanyawar, Nagrak, Sukabumi, West Java, Indonesia
C. lowi		CAS 8525	Long Mujan, Sarawak, Borneo, Malaysia
C. lowi		NHMW 17027	Baram, Miri, Sarawak, Borneo, Malaysia
C. lumbricoidea		SMF 107131	Jakata, Indonesia
C. lumbricoidea		SMF 106855	Ciwalen, West Java, Indonesi
C. lumbricoidea complex		SMF 81192	Sumatra, Indonesia
C. lumbricoidea complex		THNHM 21095	Khao Li Pae, Waeng, Narathiwat, Thailand
C. lumbricoidea complex		THNHM 21069	Khao Li Pae, Waeng, Narathiwat, Thailand
C. lumbricoidea complex		THNHM 25913	Betong, Yala, Thailand
C. lautensis	lectotype	RMNH 4716A	Cocos Is, Simeulue, Sumatra, Indonesia
C. pavimentata		SYS r001725	Nonggang NR, Guangxi, China
C. pavimentata		SYS r001726	Nonggang NR, Guangxi, China
C. pavimentata complex		THNHM 16415	Ban Doi Pri, Mae Chaem, Chiang Mai, Thailand
C. pavimentata complex		THNHM 20006	Phu Singh, Seka, Bueng Kan, Thailand
C. pavimentata complex		THNHM 15300	Sangkhla Buri, Kanchanaburi, Thailand
C. pavimentata complex		CAS 215588	Monwa, Saigaing, Myanmar
C. pavimentata complex		CAS 215546	Alaungdaw Kathapa NP, Sagaing, Myanmar
C. pavimentata complex		CAS 224575	Putao, Kachin, Myanmar

C. pavimentata complex		CAS 230226	Putao, Kachin, Myanmar
C. pavimentata complex		CAS 224647	Putao, Kachin, Myanmar
C. pavimentata complex		DTU 377	Tam Dao NP, Vinh Phuc, Vietnam
C. pavimentata complex		DTU 684	Sop Cop, Son La, Vietnam
C. pavimentata complex		DTU 595	Cau Ham Rong, Thanh Hoa, Vietnam
C. pavimentata complex		DTU 319	Bach Ma NP, Thua Thien-Hue, Vietnam
C. pavimentata complex		DTU 338	Bach Ma NP, Thua Thien-Hue, Vietnam
C. pavimentata complex		DTU 337	Bach Ma NP, Thua Thien-Hue, Vietnam
C. pavimentata complex		DTU 282	Tam Dao NP, Vinh Phuc, Vietnam
C. pavimentata complex		DTU 598	Thao Nguyen, Phieng Luong, Moc Chau, Son La, Vietnam
C. pavimentata complex		THNHM 00095	Khao Ang Rue Nai WS, Chachoengsao, Thailand
C. pavimentata complex	syntype of *C. siamensis* Günther	NHMUK 1946.1.3.40	Laos Mt
C. pavimentata complex		THNHM 00096	Khao Ang Rue Nai WS, Chachoengsao, Thailand
C. pavimentata complex		NHMW 16183	Cambodia
C. pavimentata complex		DTU 396	Dak Mil, Dak Nong, Vietnam
C. schlegeli		NHMUK 1946.1.3.44	Sinkawang, West Kalimantan, Borneo, Indonesia
C. schlegeli		NHMUK 1946.1.3.67	Sinkawang, West Kalimantan, Borneo, Indonesia
C. schlegeli		NHMUK 1946.1.3.51	Sinkawang, West Kalimantan, Borneo, Indonesia
C. schlegeli complex		NHMUK 1946.1.3.60	Sumatra, Indonesia
C. schlegeli complex		SMF 107135	Jakata, Indonesia
C. schlegeli complex		SMF 107134	Jakata, Indonesia
C. schlegeli complex		SMF 19411	Bungarbandar, Sumatra, Indonesia
C. schlegeli complex		SMF 19412	Bungarbandar, Sumatra, Indonesia
C. schlegeli complex	syntype of *C. leucocephala* Duméril, Bibron & Duméril	ZMA RENA.13805	Bungarbandar, Sumatra, Indonesia
C. schlegeli complex	syntype of *C. leucocephala* Duméril, Bibron & Duméril	RMNH 3994	Empat Lawang, South Sumatra, Indonesia
C. leucogaster		ZMA RENA 10082	Sukabumi, West Java, Indonesia
C. septentrionalis		NHMUK 1983.196	New Territories, Hong Kong, China
C. septentrionalis		NHMUK 1983.195	New Territories, Hong Kong, China
C. septentrionalis	syntype	NHMUK 1946.1.7.28	Jiujiang, Jiangxi, China
C. septentrionalis	syntype	NHMUK 1946.1.7.31	Jiujiang, Jiangxi, China
C. septentrionalis	syntype	NHMUK 1946.1.7.30	Jiujiang, Jiangxi, China
C. septentrionalis		NHBE 1017618	Ningbo, Zhejiang, China
C. septentrionalis		SMF 19438	Pingxiang, Jiangxi, China
C. septentrionalis		NHMUK 1933.11.12.2	Wuhu, Anhui, China
C. septentrionalis		NHMUK 1899.4.24.57	Wuyishan, Fujian , China
C. septentrionalis		NHMUK 1893.4.20.26	Zhoushan, Zhejiang, China
C. septentrionalis		NHMUK 1892.12.12.5	Zhoushan, Zhejiang, China

C. septentrionalis	syntype	NHMUK 1947.3.6.60	Jiujiang, Jiangxi, China
C. septentrionalis	syntype	NHMUK 1947.3.6.61	Jiujiang, Jiangxi, China
C. septentrionalis	syntype	NHMUK 1946.1.3.72	Hong Kong, China
C. septentrionalis		NHMUK 1946.1.7.29	Jiujiang, Jiangxi, China
C. septentrionalis		SMF 19437	Pingxiang, Jiangxi, China
C. septentrionalis complex		DTU 284	Phong Quang NR, Vi Xuyen, Ha Giang
C. septentrionalis complex		NHMUK 1896.4.21.1	Cao Bang, Vietnam
C. septentrionalis complex		ZMH R16944	Pingxiang, Guangxi, China
C. septentrionalis complex		ZMH R16945	Pingxiang, Guangxi, China
C. septentrionalis complex		DTU 332	Vu Quang NP, Ha Tinh, Vietnam
C. strigiventris	holotype	ZMMU R-16043	Bidoup-Nui Ba NP, Lam Dong, Vietnam
C. strigiventris	paratype	ZMMU R-16045	Bidoup-Nui Ba NP, Lam Dong, Vietnam
C. strigiventris	paratype	ZMMU R-16047	Bidoup-Nui Ba NP, Lam Dong, Vietnam
C. strigiventris		ZMMU R-11543-1	Hon Ba NR, Khanh Hoa, Vietnam
C. strigiventris		ZMMU R-11543-2	Hon Ba NR, Khanh Hoa, Vietnam
C. strigiventris	paratype	ZMMU R-16044	Bidoup-Nui Ba NP, Lam Dong, Vietnam
C. strigiventris	paratype	ZMMU R-16048	Bidoup-Nui Ba NP, Lam Dong, Vietnam
C. strigiventris	paratype	ZMMU R-16046	Bidoup-Nui Ba NP, Lam Dong, Vietnam
C. strigiventris	paratype	ZMMU R-16049	Bidoup-Nui Ba NP, Lam Dong, Vietnam
C. sumatrana		ZMA RENA.10238	Deli, North Sumatra, Indonesia
C. sumatrana		RMNH 4860	Deli, North Sumatra, Indonesia
C. sumatrana		ZMA RENA.10320	Deli, North Sumatra, Indonesia
C. sumatrana		ZMA RENA.10239	Deli, North Sumatra, Indonesia
C. sumatrana		ZMA RENA.10237	Medan, North Sumatra, Indonesia
C. synergis **sp. nov.**	holotype	ANU ZR24046	Mt. Jinuo, Jinghong, Xishuangbanna, Yunnan, China
C. synergis **sp. nov.**	paratype	ANU ZR25021	Mt. Jinuo, Jinghong, Xishuangbanna, Yunnan, China
C. virgulata		SMF 106774	Cibodas, West Java, Indonesia
C. yunnanensis		QHU R2024054	Mt. Wanzhangshan, Simao, Yunnan, China
C. yunnanensis		QHU R2024055	Mengsong, Mengla, Xishuangbanna, Yunnan, China

Suppl. Table S3. List of localities of the *Calamaria synergis* **sp. nov.**, *C. andersoni*, and *C. yunnanensis* appearing on Fig. 5. **Symbols**: (1) = Number on the map; (2) = Verified by morphology data (yes/no); (3) = Verified by molecular data (yes/no).

**(1)**	**(2)**	**(3)**	**Location**	**Sources**
***Calamaria synergis* sp. nov.**				
1	yes	yes	Mt. Jinuo, Xishuangbanna, Yunnan, China (type locality)	This study
Calamaria andersoni				
1	yes	yes	Tongbiguan, Yingjiang, Dehong, Yunnan, China (type locality)	Yang & Zheng 2018
2	yes	no	Mangdong, Lianghe, Dehong, Yunnan, China	this study
2	no	yes	Tengchong, Baoshan, Yunnan, China	Cai et al. 2023
3	yes	yes	Mangshi, Dehong, Yunnan, China	Cai et al. 2023; this study
Calamaria yunnanensis				
1	yes	no	Jingdong, Pu'er, Yunnan, China (type locality)	Lee et al. 2021
2	yes	yes	Mt. Wanzhangshan, Simao, Yunnan, China	Lee et al. 2021; this study
3	yes	no	Mengsong, Mengla, Xishuangbanna, Yunnan, China	this study
